# Artificial intelligence in biomedical visualization and education: a narrative review

**DOI:** 10.3389/fpubh.2026.1956708

**Published:** 2026-09-29

**Authors:** Xin Wang, Linlin Song, Bolin Liu, Jie Chen, Haina Zhao

**Affiliations:** 1 Department of General Surgery, Division of Breast Surgery, West China Hospital, Sichuan University, Chengdu, China; 2 Breast Center, West China Hospital, Sichuan University, Chengdu, China; 3 Ultrasound Department, Institute of Breast Health Medicine, West China Hospital, Sichuan University, Chengdu, China; 4 Department of Operations Management, West China Hospital, Sichuan University, Chengdu, China

**Keywords:** 3D reconstruction, artificial intelligence, biomedical visualization, medical education, medical illustration, vision-language models

## Abstract

Biomedical visualization increasingly spans traditional medical illustration, synthetic medical imaging, vision-language systems, and patient-specific 3D reconstruction. This narrative review examines applications of artificial intelligence (AI) across this field, with particular attention to medical education and scientific and clinical communication. We searched PubMed/MEDLINE and IEEE Xplore for English-language literature published from January 2017 through September 2026, supplemented by targeted searches of publisher databases and reference-list screening. Included publications addressed AI-enabled medical or anatomical illustration, text-to-image generation, synthetic medical imaging, vision-language models, 3D reconstruction, or immersive biomedical visualization. Current evidence indicates that diffusion models can rapidly generate visually persuasive and customizable drafts, whereas segmentation-guided synthesis and anatomy-aware 3D methods offer stronger structural control in defined tasks. However, direct evaluations of general-purpose text-to-image systems consistently identify fabricated anatomy, incorrect labels, prompt- and model-dependent variability, demographic bias, and limited reliability for complex structures or procedures. Vision-language models can support prompt refinement, annotation, and consistency checking, but they cannot ensure scientific accuracy and may evaluate flawed AI outputs more favorably than human experts. Evidence supporting AI-generated educational illustrations remains less mature than that supporting conventional 3D and virtual-reality learning environments. We therefore distinguish established applications from emerging or prospective uses and propose an expert-supervised workflow incorporating documented prompts and model versions, structure-specific validation, manual correction, and transparent disclosure. AI should currently be treated as a drafting and visualization aid, with domain-expert verification required before educational, clinical, or scientific use.

## 1 Introduction

Visual representations help learners and clinicians understand anatomy, disease mechanisms, clinical procedures, and patient-specific findings. Professional medical illustrators integrate scientific interpretation with deliberate choices regarding viewpoint, abstraction, labeling, and visual hierarchy. Although digital tools have accelerated production, high-quality medical illustrations still require subject-matter expertise, iterative refinement, and expert review (1, 2).

Generative artificial intelligence (AI) has introduced additional methods for producing biomedical visual content. Diffusion models can generate images from text, sketches, masks, or reference images (3, 4), while biomedical vision-language models integrate visual inputs with domain-specific text (5). Task-specific systems can also synthesize radiologic images and reconstruct three-dimensional anatomy. These approaches serve different purposes and should not be evaluated using a single standard. Anatomical illustrations, synthetic chest radiographs, and reconstructed surgical fields have distinct error modes and require task-specific validation.

This narrative review examines the principal AI approaches used in biomedical visualization, summarizes direct evaluations of AI-generated medical and anatomical illustrations, and distinguishes evidence-supported applications from proposed uses. It also considers training data, reproducibility, bias, copyright, and expert oversight. The central question is whether each output is sufficiently accurate and reliable for its intended biomedical use.

### 1.1 Literature search and study selection

A structured literature search was conducted in PubMed/MEDLINE and IEEE Xplore for publications from January 2017 to September 2026. Additional targeted searches were performed in SpringerLink, Wiley Online Library, ScienceDirect, and JMIR Publications to identify relevant studies not captured in the primary database searches. The search combined terms for AI methods (“artificial intelligence,” “generative AI,” “text-to-image,” “diffusion model,” “generative adversarial network,” “vision-language model,” and “3D reconstruction”) with visualization terms (“medical illustration,” “anatomical illustration,” “biomedical visualization,” “medical image synthesis,” “medical education,” “patient education,” and “scientific visualization”). Reference lists of relevant reviews and original studies were also screened.

We included original studies, reviews, and peer-reviewed conference papers that addressed the AI-enabled generation, reconstruction, evaluation, or application of biomedical visual content. Particular emphasis was placed on studies that directly evaluated anatomical or scientific accuracy, prompt effects, expert ratings, educational utility, bias, or reproducibility. We excluded purely diagnostic classification studies without a generative or visualization component, general-purpose image-generation studies without biomedical evaluation, duplicate publications, and non-peer-reviewed promotional reports.

The primary database searches retrieved 3,841 records from PubMed/MEDLINE and 628 records from IEEE Xplore, yielding 4,469 records before cross-database deduplication. The final narrative synthesis drew on 40 substantive publications, of which 15 original studies met the predefined criteria for inclusion in Table 1 as direct evaluations of AI-generated medical or anatomical images.

**Table 1:** Direct evaluations of AI-generated medical and anatomical images.

Study	Models and domain	Validation approach	Main finding
Noel, 2024 (15)	Three generators; skull, heart, and brain	Anatomical detail and accuracy review	No generator was consistently both detailed and accurate
Kumar et al., 2024 (16)	DALL-E 2 and Midjourney; hypothyroidism and Horner syndrome	Clinician-led iterative prompting and visual review	Symmetric features were easier; asymmetric signs required manual editing
Buzzaccarini et al., 2024 (17)	Midjourney; systemic anatomy and esthetic procedures	Blinded panel of four anatomy and surgery experts	High visual impact but substantial anatomical inaccuracy
Temsah et al., 2024 (18)	DALL-E 3; 110 normal and congenital-heart images	33 health professionals; 3,630 ratings	80.8% incorrect or fabricated; 78.1% not usable for education
Moin et al., 2024 (12)	DALL-E 3 and MIM; corneal transplant procedures	Four independent reviewers, human controls, and ChatGPT-4o grading	Human controls scored 97.1% versus 29.2 and 37.5% for AI outputs; AI grading was overly favorable
Petroff et al., 2024 (19)	DALL-E 3 and MiM; LASIK, PRK, and SMILE	Four corneal surgeons; human controls; five-domain rubric; ChatGPT-4o re-rating	Human controls scored 95.6% versus 32.8 and 38.9% for AI outputs; ChatGPT-4o overrated AI images
Muhr et al., 2024 (20)	DALL-E 3, Stable Diffusion XL, and Stable Cascade; photorealistic human anatomy	240 images; 10 prompts; five error types and three severity levels; 25% double-annotated	Errors varied by model and prompt; groups and hands were difficult, and inter-rater agreement was limited
Haider et al., 2025 (21)	Four models; 736 craniofacial images	Four reviewers; anatomical, esthetic, usability, and reliability measures	Strong inter-rater reliability, but all models failed on important complex details
Eldesoqui et al., 2025 (22)	Four models; heart, brain, thorax, and hand	Two anatomists and one radiologist	Model and structure affected accuracy; bone detail was a recurrent weakness
Bae et al., 2025 (23)	DALL-E 2, Midjourney 5, and Stable Diffusion 1.5; later DALL-E 3 and Midjourney 6; bones, organs, and muscles	Anatomical terminology and accuracy comparison, followed by evaluation of updated model versions	Newer versions improved image quality, but professional medical illustrator expertise remained necessary
Davis et al., 2025 (24)	Four models; neurovascular anatomy and procedures	Standardized rubric; simple versus advanced prompts; trainee and attending validation	Advanced prompts improved scores, but complex anatomy remained unreliable
Yang et al., 2025 (25)	Sora Turbo; anterior-segment images	Ophthalmologists with different experience levels	Gross pathology was more plausible than fine structure; senior raters were more critical
Paslı et al., 2025 (26)	Four generators; visceral, cadaveric, and genital anatomy	Two evaluators; nine criteria for anatomy, educational utility, and visual quality	ImageFX scored highest and Craiyon lowest; visually appealing images still lacked important anatomical features
Strugala et al., 2026 (27)	Six generators; multiple anatomical structures	Standardized prompt and multi-rater structure-level analysis	Large variation across anatomy and models; expert verification remained necessary
Albadawi et al., 2026 (28)	Copilot, Microsoft Designer, Gemini, and Freepik; heart, kidney, and brain	12 images; one radiologist and two anatomists; five-domain 5-point Likert assessment	Gemini performed best on complex topology, but accuracy declined with structural complexity; expert supervision remained necessary

MIM, Medical Illustration Manager/Master as named by the source study. The studies use different prompts, model versions, sampling strategies, and rating scales, so their numerical results should not be compared as a pooled benchmark.

### 1.2 Review scope and terminology

This review covers three principal forms of AI-enabled biomedical visual content: AI-generated explanatory or anatomical illustrations; synthetic clinical images used for visualization, model training, or data augmentation; and AI-assisted reconstruction of patient-specific three-dimensional anatomy. It examines the use of these outputs in medical education, clinical communication and patient education, research visualization and scientific publishing, and public health communication.

Quantitative data visualization and diagnostic AI are considered only when they directly inform visual generation or validation. Conventional virtual-reality and augmented-reality studies are included only as evidence for the educational value of immersive delivery; they are not treated as evidence that AI-generated three-dimensional content is anatomically accurate or educationally effective.

## 2 AI technologies for biomedical visualization

### 2.1 Image generation from GANs to diffusion models

Generative adversarial networks (GANs) established many early medical image-synthesis applications. They have been used for modality translation, data augmentation, and simulation of radiologic or histologic appearances (6). Examples include unpaired MR-to-CT synthesis and cross-modal cardiac image synthesis (7, 8). These studies support image translation within constrained medical datasets, but they do not establish that GANs can independently create accurate explanatory diagrams. Training instability, mode collapse, and limited semantic control also restrict their use for complex labeled illustrations.

Diffusion models generate images through iterative denoising and provide more flexible text or image conditioning than earlier GAN pipelines (3, 4). General-purpose systems can produce rapid visual drafts and stylistic variants, but photographic realism and prompt adherence are not measures of anatomical correctness. Domain adaptation can improve performance in defined tasks. For example, RoentGen generated text-conditioned chest radiographs after medical image-text fine-tuning, with radiologist-based and computational evaluation (9). Segmentation-guided diffusion can enforce selected anatomical masks in breast MRI and CT synthesis (10), while MedSyn combines radiology text with airway, vascular, and lobar structure conditioning for 3D lung CT generation (11). These systems show that explicit structural constraints are more reliable than unconstrained prompting for task-specific synthesis. Their performance should not be generalized to textbook-style illustration without direct evaluation.

### 2.2 Vision-language models

#### 2.2.1 Annotation and prompt support

VLMs align images with text and can support captioning, terminology suggestions, image-based question answering, prompt refinement, and comparison between a requested concept and a generated image. Biomedical systems such as LLaVA-Med demonstrate the feasibility of instruction tuning on biomedical image-text data (5). Performance varies according to the domain, task, prompt, and quality of the visual input. Fluent labels or explanations do not establish that the underlying anatomy is correct.

#### 2.2.2 Knowledge grounding and output verification

Structured terminologies such as UMLS and RadLex may improve consistency in prompts and labels. However, no study identified in this review demonstrated that integrating these resources with a general-purpose VLM reduces anatomical errors. In contrast, task-specific generative systems have achieved more direct structural control through paired medical image–text data and spatial conditioning, including segmentation masks and anatomical landmarks (9–11). VLMs can assist with semantic consistency checks and flag obvious mismatches, but they cannot guarantee anatomical or scientific accuracy. In a study of corneal transplant illustrations, ChatGPT-4o scored AI-generated images substantially more favorably than independent human reviewers, showing that automated evaluation may itself miss or underweight important errors (12). Final validation by an appropriately qualified human expert therefore remains necessary.

### 2.3 AI-assisted 3D generation and reconstruction

AI-based 3D methods include volumetric diffusion, mesh generation, and neural radiance fields (NeRFs). Their strongest evidence currently concerns reconstruction or synthesis from imaging data rather than free-form generation of educational anatomy. MedNeRF reconstructed 3D-aware CT projections from few or single-view radiographs in chest and knee datasets (13). A cadaveric sinus-surgery study used NeRF to reconstruct a surgical field from monocular endoscopic video and reported submillimeter performance relative to postoperative CT (14). MedSyn generated high-resolution 3D lung CT volumes conditioned on text and anatomical structures (11). These studies demonstrate the potential of AI-assisted 3D visualization, but their findings remain specific to particular inputs, anatomical regions, and validation tasks.

Despite these technical advances, reconstruction accuracy should not be equated with educational effectiveness. Generated or reconstructed assets may be incorporated into 3D software, simulators, or VR/AR environments, but evidence of geometric or imaging fidelity does not demonstrate that the resulting learning environment improves knowledge, spatial understanding, or procedural skills. Such claims require direct evaluation involving learners.

More broadly, these examples illustrate a recurring trade-off across model classes. Source images, segmentation masks, landmarks, and explicit geometry generally provide greater structural control and enable more objective validation, but their performance may not transfer beyond the specific tasks and datasets for which they were developed. In contrast, general-purpose text-to-image models offer greater flexibility but provide less reliable control over topology, laterality, labeling, and procedural sequence. The key distinction is therefore the degree of structural constraint and task-specific validation, rather than simply the novelty of the model. Table 2 summarizes these differences and their corresponding validation requirements.

**Table 2:** Comparison of major AI technologies and evidence maturity in biomedical visualization.

Technology	Main application	Potential advantage	Main limitation	Validation and current evidence
GAN-based medical image synthesis (6–8)	Modality translation and data augmentation	Mature training pipelines for constrained datasets	Training instability; weak prompt-level control; realism does not establish illustration accuracy	Image similarity, downstream-task performance, and specialist review; established for selected imaging tasks, limited for explanatory illustration
General-purpose text-to-image diffusion (3, 4, 15–28)	Draft anatomical, procedural, or patient-facing visuals	Fast generation, broad style range, easy iteration	Fabricated anatomy and labels; variable output across models, prompts, and runs	Expert rubrics and repeated generation; emerging and not independently reliable
Domain-adapted or structure-guided diffusion (9–11)	Synthetic radiographs and anatomy-aware 3D volumes	Better text alignment and spatial control within a defined domain	Dependent on curated data and explicit masks; uncertain transfer to schematic education	Radiologist review, mask adherence, image metrics, and downstream tasks; established only in bounded tasks
Vision-language models (5, 12)	Captioning, prompt refinement, terminology support, and consistency checks	Connect visual and textual context	May hallucinate or overrate flawed AI outputs; cannot certify correctness	Agreement with human experts, error analysis, and test–retest checks; supportive rather than definitive
NeRF and AI-assisted 3D reconstruction (13, 14)	Patient-specific and surgical-field reconstruction	Novel-view rendering and spatial representation from limited views	Occlusion, deformation, domain shift, and uncertain educational transfer	Geometric error, comparison with CT or tracked anatomy, and task-specific expert review; emerging clinical evidence
VR and AR delivery environments (29–31)	Interactive anatomy and simulation	Supports manipulation and spatial learning	Benefits depend on instructional design; studies often do not use AI-generated assets	Learner outcomes and usability; established as a delivery modality, AI-content generation remains emerging
Human-AI co-creation	Expert-directed drafting, correction, labeling, and publication preparation	Combines production speed with domain judgment	Requires reviewer time, version control, and clear responsibility	Documented prompts, expert checklist, manual correction, and disclosure; currently the safest operational model

AI, artificial intelligence; AR, augmented reality; GAN, generative adversarial network; NeRF, neural radiance field; VR, virtual reality. Evidence maturity refers to the specific biomedical visualization use shown, not to the general maturity of the underlying model.

## 3 Applications and current evidence

Technical capability does not by itself establish educational or clinical value. The relevant endpoint changes with the application: anatomical fidelity and learning outcomes for education; correspondence with source imaging and comprehension for patient communication; scientific validity and provenance for publishing; and message comprehension for public communication. The following sections therefore interpret evidence according to the intended user, decision, and consequence of error.

### 3.1 Medical education

Text-to-image systems can help educators create alternative viewpoints, vary visual complexity, or prototype case-based materials. They may be useful when an illustration is treated as a draft that will be compared with an authoritative source and corrected. They are less suitable when learners may interpret an unverified image as canonical anatomy. Current direct evidence is dominated by model-evaluation studies rather than trials showing improved learning outcomes.

#### 3.1.1 Direct evaluations of anatomical accuracy and educational reliability

Several studies now directly test AI-generated medical illustrations. Noel et al. evaluated three text-to-image generators for the skull, heart, and brain and found that none consistently produced images that were both detailed and anatomically accurate (15). Kumar et al. could generate recognizable symmetric facial features of hypothyroidism, whereas depicting asymmetric Horner syndrome required manual editing (16). In esthetic surgery, expert reviewers found high visual impact but substantial anatomical inaccuracy in Midjourney outputs (17).

More structured evaluations show the same gap between visual appeal and educational fitness. Temsah and colleagues generated 110 DALL-E 3 images of a normal heart and congenital heart diseases; 80.8% were judged anatomically incorrect or fabricated, 85.2% had incorrect in-image text, and 78.1% were considered unusable for medical education (18). Human-created corneal procedure illustrations also substantially outperformed DALL-E 3 and a specialized illustration system, while ChatGPT-4o rated the AI outputs more favorably than human reviewers (12, 19). A systematic error taxonomy applied to 240 photorealistic human images generated by DALL-E 3, Stable Diffusion XL, and Stable Cascade showed model- and prompt-dependent differences, with group scenes proving especially difficult and inter-rater agreement remaining limited (20).

Recent studies broadened these findings. In a 736-image craniofacial analysis, DALL-E 3 and Midjourney performed better than the other tested systems, but all models made important errors in foramina, sutures, muscle attachments, neurovascular structures, and labels (21). Evaluations of heart, brain, thorax, and hand images also found substantial differences among platforms and poorer performance for complex bony detail (22). Bae and colleagues compared DALL-E 2, Midjourney 5, and Stable Diffusion 1.5, with a follow-up evaluation of DALL-E 3 and Midjourney 6. Newer versions improved image quality, but the authors still supported complementary rather than autonomous use (23). Advanced prompting improved ratings in a multistage neurosurgical study, yet complex vascular anatomy remained less accurate and less educationally useful (24). Ophthalmology evaluation similarly found that gross appearances could look plausible while fine structures remained unreliable (25). Paslı et al. compared ChatGPT, Microsoft Designer, Craiyon, and ImageFX for visceral, cadaveric, and genital anatomy. Two researchers independently scored nine criteria covering anatomical features, educational utility, and visual quality. ImageFX achieved the highest overall score and Craiyon the lowest, but visually appealing outputs still omitted important anatomical features, and platform content restrictions affected some anatomical promptes (26). Studies published in 2026 continue to report marked model- and region-specific variability and recommend expert verification (27, 28).

Taken together, these studies identify recurring anatomical and labeling errors, particularly in complex structures and procedures. However, differences in anatomical topics, prompts, model versions, and evaluation methods preclude pooled accuracy estimates or stable platform rankings. Visual plausibility alone should therefore not be considered evidence of anatomical accuracy or educational suitability. For transparency, Table 1 includes all eligible original studies identified through the review search that systematically evaluated AI-generated medical or anatomical images using expert ratings or a structured anatomy-focused evaluation framework. Conceptual reports, illustrative case examples without systematic evaluation, and studies limited to conventional VR/AR delivery were not included in Table 1.

#### 3.1.2 Immersive and interactive learning

Interactive 3D, VR, and AR environments can improve spatial exploration and learner engagement. A randomized study of a computer-generated 3D ear model and later reviews of immersive anatomy education support the educational potential of these delivery formats (29–31). These publications did not evaluate AI-generated 3D anatomy. Claims about AI-driven reconstruction should therefore be supported by geometric or imaging validation (11, 13), while claims about learning should be tested with knowledge, retention, transfer, or procedural outcomes.

### 3.2 Clinical communication and patient education

Patient-specific visuals may help explain lesion location, anatomy, or a planned procedure when they are derived from verified patient imaging and corrected by the treating team. AI-assisted reconstruction can reduce manual modeling time, but it does not replace review of the source CT, MRI, ultrasound, or endoscopic data. Any image used for consent or decision-making should preserve clinically relevant anatomy, clearly distinguish confirmed findings from hypothetical content, and avoid adding structures that are absent from the source.

Simplified visual materials may also support people with limited health literacy. Visual interventions generally improve some health-literacy outcomes, although effects vary by design and context (32). This evidence supports careful visual communication, not the assumption that AI-generated graphics are effective. Patient-facing AI images should be evaluated for comprehension, risk perception, and decisional quality in the intended population.

### 3.3 Research visualization and scientific publishing

Generative tools can accelerate early drafts of graphical abstracts, mechanism diagrams, and workflow illustrations. They are most useful for composition, style exploration, or background elements. Directed arrows, molecular relationships, labels, numerical values, and scale require manual construction or verification. VLM-assisted checking may detect some prompt-image inconsistencies, but it cannot certify the scientific logic of a pathway or the provenance of a visual element (12).

AI-generated radiologic or histologic images can also support method development and data augmentation in bounded research settings. The validation standard should match the claim: image realism for visual simulation, anatomical fidelity for reconstruction, downstream-task performance for augmentation, and reader studies for clinical or educational use (9–11). Authors should report the model and version, prompts or conditioning inputs, sampling settings when available, post-processing, and the identity or expertise of reviewers.

### 3.4 Public health and digital outreach

Generative systems have been proposed for multilingual infographics, social-media content, and public-health campaign drafts. However, this review did not identify adequate evidence that AI-generated COVID-19 transmission, vaccination, or prevention graphics improved comprehension or behavior. We therefore regard public-health graphics as a prospective application rather than an established use. Public-facing outputs require content verified against authoritative sources, audience testing, accessibility checks, and expert approval, particularly when visual simplification could alter risk communication (32, 33).

Future studies should compare AI-assisted materials with professionally produced graphics using prespecified outcomes, including comprehension, recall, risk perception, accessibility, and behavior (33). They should also assess whether demographic representation affects audience trust or interpretation.

## 4 Training data and model development

### 4.1 Data quality, provenance, and representation

General image datasets contain relatively little high-quality, consistently labeled biomedical illustration, while textbooks, atlases, journal figures, pathology slides, and surgical images carry copyright, licensing, privacy, and provenance constraints. For the applications reviewed here, this creates an uneven training base: common external anatomy and stereotyped patient depictions may be overrepresented, whereas rare disease, internal spatial relationships, anatomical variation, and underrepresented populations remain sparse (34, 35). Dataset composition and source disclosure therefore affect both model capability and the credibility of downstream validation.

Beyond data availability, anatomical illustration is difficult to annotate because one image may encode multiple scales, tissue layers, viewpoints, variants, and causal relationships. Labels also differ across specialties and institutions. Domain terminologies can improve naming consistency, but they cannot by themselves define shape, topology, laterality, or clinical relevance. Curated datasets should therefore combine controlled terms with landmarks, masks, relational annotations, source citations, and explicit information about normal variation.

Representation must be audited as part of data quality. Large-scale analysis of three text-to-image generators found demographic imbalance in surgical depictions (34). In dermatology, 89.8% of 4,000 generated images depicted lighter skin and only 15% of a rated subset accurately represented the intended condition (36). Representation gaps also occur in conventional anatomy atlases and textbooks (35), so a curated AI dataset should not reproduce those historical omissions.

### 4.2 Domain adaptation and structural control

Low-rank adaptation, full fine-tuning, and retrieval or reference-image conditioning can make general models more domain specific. Structure-guided methods provide more direct constraints. Segmentation-guided diffusion can condition generation on selected anatomical classes (10), and MedSyn jointly uses text and anatomical masks to preserve lung structures in 3D CT synthesis (11). These approaches support the claim that explicit spatial conditioning can improve structural adherence in a defined imaging task. They do not justify the stronger claim that ontology-guided generation already prevents errors in general medical illustration.

## 5 Quality assessment and responsible use

### 5.1 Fitness for purpose

Quality should be defined by the intended use rather than esthetic appeal. An educational anatomy image requires correct structures, spatial relationships, laterality, scale, variants, and labels. A mechanism diagram requires correct entities, directionality, compartmentalization, and causal qualification. A patient-specific model requires correspondence with source imaging and clinically important boundaries. A public-health graphic requires accurate content, comprehension, accessibility, and absence of misleading emphasis.

The direct evaluation literature supports multi-domain rubrics that separate visual quality from anatomical accuracy, educational utility, labeling, and fabricated content (12, 17–25, 27). Independent expert review and inter-rater agreement should be reported where possible. Automated image metrics may measure similarity or distributional quality in synthetic imaging, but they do not substitute for structure-specific or clinical assessment (9–11, 13, 37).

### 5.2 Reproducibility, bias, and safety

Text-to-image outputs vary with model version, prompt wording, negative prompts, reference images, random seed, inference settings, and provider-side updates. Davis and colleagues showed that advanced prompting materially improved scores for several neurosurgical topics, whereas Muhr and colleagues found prompt- and scene-dependent error patterns (20, 24). Reproducible reporting should include the platform, model and version, access date, complete prompt, number of attempts, selection process, editing steps, and reviewer procedure. Images chosen from many unreported attempts can otherwise exaggerate apparent reliability.

Bias and safety also require specific assessment. Inaccurate illustrations may teach false spatial relationships, distort informed consent, or misrepresent particular populations. Bias can arise during problem definition, data selection, labeling, model training, prompting, output selection, or deployment; evaluation should therefore include appropriate subgroup and representation analyses (33). Patient-derived inputs must also be de-identified and managed under appropriate data-governance procedures, because source images may contain identifiable or sensitive information even when the final output is schematic.

### 5.3 Authorship, copyright, and disclosure

Medical images have ethical histories and obligations that extend beyond technical accuracy. The International Federation of Associations of Anatomists has issued recommendations for the ethical use of anatomical images (38). For AI-generated work, authors should document source permissions, disclose material AI assistance, and remain accountable for every scientific claim and visual element. Copyright protection for predominantly machine-generated content remains uncertain and depends on jurisdiction and the extent of human authorship; legal scholarship and current policy guidance emphasize the importance of identifiable human creative contribution (39).

## 6 Human-AI co-creation and validation

The evidence supports a supervised workflow rather than autonomous generation. First, a clinician, educator, scientist, or medical illustrator defines the learning or communication objective and lists the structures or relationships that must be correct. Second, the creator records the model, version, prompt, reference material, and generation settings. Third, several drafts are compared with an authoritative source. Fourth, a qualified reviewer checks structure, labels, relationships, variants, omissions, and audience suitability. Fifth, the selected image is corrected in a professional graphics or 3D tool. The final version is approved, archived with its provenance, and disclosed as AI-assisted when required.

This workflow assigns AI the tasks it performs well, such as rapid drafting and variation, while retaining human responsibility for scientific interpretation. Human-AI collaboration can improve performance in other medical decision tasks, but its benefit depends on calibrated trust and appropriate allocation of responsibility (40). In biomedical visualization, reviewers should be alert to visually persuasive errors and to automation bias when an AI system evaluates another AI system (12).

## 7 Research priorities

The field needs shared, versioned benchmarks containing normal anatomy, variants, procedures, pathology, and diverse patient representations. Evaluation should include topology and laterality, fine structures, label fidelity, prompt robustness, run-to-run variation, and the number of attempts required to obtain an acceptable image. Studies should use blinded expert review and report inter-rater reliability. Learner and patient studies should measure comprehension, retention, transfer, risk perception, and decision quality rather than relying only on perceived attractiveness or usefulness.

Knowledge-grounded models are a promising direction, but future work should test whether ontologies, retrieval from curated sources, scene graphs, masks, and landmark constraints improve factual accuracy beyond unconstrained baselines. For 3D reconstruction, studies should separate geometric fidelity, visual realism, clinical utility, and educational effect. Prospective comparisons with professionally produced illustrations and multicenter validation across specialties would provide stronger evidence for deployment.

## 8 Limitation

This review has several limitations. First, PubMed/MEDLINE and IEEE Xplore were the primary databases searched. Although these searches were supplemented by targeted searches of publisher databases and reference-list screening, relevant studies indexed elsewhere may have been missed, introducing potential selection bias. Second, eligibility was restricted to English-language publications, which may have introduced language bias and limited the representation of evidence from non-English-speaking settings. Third, the narrative review design did not employ a prespecified systematic-review protocol or a uniform study-level risk-of-bias instrument across the heterogeneous study designs. Differences in technologies, prompts, model versions, application domains, evaluation methods, and outcome measures also precluded quantitative pooling and limit direct comparisons between studies. In addition, the rapid evolution of commercial and research models makes individual performance estimates and platform rankings time-sensitive. Finally, because this review covers multiple medical specialties, it can identify common strengths and limitations of AI-generated biomedical images but cannot provide detailed conclusions for each specialty. The findings should therefore be regarded as an overview of the current evidence, which may change as AI models evolve, rather than as a basis for ranking models or making clinical decisions.

## 9 Conclusion

AI has widened the production methods available for biomedical visualization. General-purpose text-to-image models are fast and visually persuasive, while domain-specific synthesis and 3D reconstruction systems can provide stronger control in bounded tasks. Direct studies nevertheless show frequent anatomical errors, incorrect labels, prompt-dependent variability, and demographic bias, especially for complex structures and procedures. VLMs can support annotation and checking but cannot ensure correctness. Conventional VR and AR studies support immersive learning, but they do not validate AI-generated 3D assets. The safest current use is expert-supervised co-creation with documented inputs, explicit validation, manual correction, and transparent disclosure. Wider educational or clinical adoption should depend on evidence that outputs are accurate for their intended purpose and effective for their intended audience.
